# Status Epilepticus due to Intraperitoneal Injection of Vehicle Containing Propylene Glycol in Sprague Dawley Rats

**DOI:** 10.1155/2017/1757059

**Published:** 2017-07-12

**Authors:** Evon S. Ereifej, Seth M. Meade, Cara S. Smith, Keying Chen, Nanette Kleinman, Jeffrey R. Capadona

**Affiliations:** ^1^Advanced Platform Technology Center, Louis Stokes Cleveland Department of Veterans Affairs Medical Center, 10701 East Blvd, 151 W/APT, Cleveland, OH 44106, USA; ^2^Department of Biomedical Engineering, Case Western Reserve University, 2071 Martin Luther King Jr. Drive, Wickenden Bldg., Cleveland, OH 44106, USA

## Abstract

Published reports of status epilepticus due to intraperitoneal injection containing propylene glycol in rats are sparse. In fact, there are no reports specifying a maximum safe dose of propylene glycol through intraperitoneal administration. We report here a case of unexpected seizures in Sprague Dawley rats after receiving an intraperitoneal injection containing propylene glycol. Nine-week-old, 225–250 gram male rats were reported to experience tremor progressing to seizures within minutes after given injections of resveratrol (30 mg/kg) dissolved in a 40 : 60 propylene glycol/corn oil vehicle solution by direct intraperitoneal (IP) slow bolus injection or via a preplaced intraperitoneal catheter. The World Health Organization suggests a maximum dose of 25 mg/kg/day of propylene glycol taken orally and no more than 25 mg/dL in blood serum, whereas the animals used in our study got a calculated maximum 0.52 g/kg (25 times lower dose). Blood tests from the seizing rat support a diagnosis of hemolysis and lactic acidosis which may have led to the seizures, all of which appeared to be a consequence of the propylene glycol administration. These findings are consistent with oral and intravenous administration of propylene glycol toxicity as previously reported in other species, including humans. To our knowledge, this report represents the first published case of status epilepticus due to an IP injection containing propylene glycol.

## 1. Introduction

Propylene glycol is a clear, colorless, odorless compound commonly used in pharmaceuticals, cosmetics, and food [1, 2]. Several studies have shown propylene glycol is safe and not toxic [2–6]. Specifically studying rats, Gaunt et al. performed toxicological studies by feeding Charles River CD strain Sprague Dawley rats with diets containing 0–50,000 ppm (equivalent to 2–5 g/kg/day) propylene glycol for two years. They observed no differences in mortality, body-weight gain, food consumption, hematology, urinary cell excretion, or any other pathological findings between controls and groups fed with propylene glycol in their diets [4]. Years later in 1982, the Food and Drug Administration classified propylene glycol as “generally regarded as safe” [1]. Furthermore, the World Health Organization suggests a maximum dose of 25 mg/kg/day of propylene glycol taken orally and no more than 25 mg/dL in blood serum [1, 7, 8]. To date, there is no information on the bioavailability or toxicity of propylene glycol delivered intraperitoneally.

This report discusses a case in which propylene glycol was used to dissolve resveratrol for IP slow bolus administration to rats. Propylene glycol has been used to dissolve resveratrol in several studies, some of which safely used up to 50% propylene glycol in a rodent model [9, 10]. The majority of published warnings against the use of propylene glycol as a vehicle for insoluble drugs are for epileptic studies and human patients [11–13]. Studies have shown propylene glycol can influence seizures in animal models of epilepsy and warn against using propylene glycol as the solvent to dissolve epileptic drugs such as carbamazepine, trimethadione, and benzodiazepines [2, 3, 11, 13]. Recent clinical data has shown toxicity of propylene glycol secondary to lactic acidosis, resulting from the metabolism of propylene glycol, after intravenous administration of an antibiotic medication, trimethoprim-sulfamethoxazole, which contains propylene glycol [14]. We report here a case of unexpected seizures in Sprague Dawley rats after receiving an intraperitoneal injection of resveratrol in a 40 : 60 propylene glycol/corn oil vehicle solution.

## 2. Case Presentation

Nine-week-old, 225–250 gram male rats were reported to experience tremor progressing to seizures within minutes after given slow bolus intraperitoneal (IP) injections of sterile resveratrol (30 mg/kg; Mega Resveratrol, Candlewood Stars Inc.) dissolved in 40 : 60 propylene glycol/corn oil (Sigma Aldrich) vehicle solution (equivalent to 0.52 g/kg of propylene glycol). The Sprague Dawley rats were part of an IACUC approved protocol. The rats were housed at the Louis Stokes Cleveland Veteran Affairs Medical Center in AAALAC-accredited animal research facilities. Upon arrival, rats were housed in a temperature (21 ± 2°C) and humidity (30% to 70%) controlled room with a 12 : 12 hr light : dark cycle. Standard rodent chow (Teklad irradiated 7912 rat diet, Harlan Teklad, Madison, WI) and autoclaved reverse-osmosis–purified water supplied by water bottle were freely available. Sprague Dawley rats were monitored quarterly by sentinels housed in the same room which were documented to be free of viruses (Charles River Research Animal Diagnostic Services Rat Tracking Profile), mites, pinworms (PCR), and enteric protozoa and helicobacter spp. (PCR).

The Sprague Dawley rats reported on in this manuscript are a part of an ongoing drug study evaluating the use of resveratrol to reduce neurodegeneration and improve intracortical microelectrode recording quality. At the time of incidence, there were another five animals ongoing with the study receiving resveratrol every other day for up to 7 weeks without any complications. All animals underwent craniotomies in which Michigan style intracortical microelectrodes (NeuroNexus, Ann Arbor, Michigan) were implanted into the primary motor cortex. Animals received a preoperative injection of resveratrol intraperitoneally (traditional IP slow bolus injection with a needle going either directly to the IP cavity or through an interscapular port implanted subcutaneously and connected to a catheter sutured in the muscle wall of the IP cavity) 16 hours before surgery. It is important to note that the type of IP injection (with or without a catheter) did not influence the outcomes observed; all animals receiving propylene glycol in their injections experienced a seizure.

During the preoperative injections (300 *µ*L vehicle with 120 *µ*L being propylene glycol), it was found that the rats began to have seizures within 2 minutes ± 1 minute. Presented in this case report is a series of retrospective case reports, describing seven rats that were evaluated in order to understand the acute toxicity occurring after the resveratrol administration (Table 1). Postmortem necropsy analysis was performed on the first rat that seized and died after resveratrol administration (*n* = 1). All postmortem findings were normal. No gross abnormalities were identified at necropsy leading to a diagnosis of death due to status epilepticus. Etiology of seizure was undetermined but thought to be secondary to toxicity from resveratrol or diluent. There were no gross signs of abdominal inflammation, infection, or organ dysfunction secondary to injections through injection port. Ingesta in stomach and normal organs supports diagnosis of acute/peracute death without preexisting pathology. The lung lesions found were likely agonal in origin.

A second rat experienced status epilepticus following resveratrol administration. Since this was the second rat to experience seizures following administration of the drug, it was hypothesized that the animals could be suffering from seizures due to hypoglycemia resulting from the metabolism of the propylene glycol diluent in the drug. Therefore, 50% dextrose was administered to the seizing animal in order to diagnose the hypoglycemia and potentially prevent future seizures. It was found that 50% dextrose delivered orally (0.5 mL) or rectally (1.0 mL) within two minutes of initial tremors was able to stop the seizures 3 ± 2 minutes from dextrose administration time in 80% of the rats. Depending on the severity of the tremors, some of the animals were unable to receive the dextrose orally and would need to have it administered rectally.

Blood glucose levels were measured from either the tail or toe nail of one rat that had not received any prior resveratrol injections (*n* = 1). Healthy rat blood glucose levels are measured to be 50–135 mg/dL. The blood glucose level of the rat prior to resveratrol injections was measured to be 125 mg/dL. At the time of initial seizing, blood glucose levels dropped to 106 mg/dL; however they kept rising during the seizing episode, reaching a stable level of 165 mg/dL. Administration of 50% dextrose orally and rectally was able to stop the seizures and a final blood glucose level of 236 mg/dL was measured once the animal had recovered. The results from the glucose tests indicated the animals were not hypoglycemic and the activation of the seizures was due to the compound being injected.

To understand which of the substances in the drug compound was the cause of the seizures, tests were performed evaluating the diluent, as well as each separate component of the diluent. When either the diluent, 40 : 60 propylene glycol/corn oil, (*n* = 1) or 100% propylene glycol (*n* = 1) was administered intraperitoneally to the rats, the animals began to seize within 1.5 minutes. However, when 100% corn oil (*n* = 1) was injected intraperitoneally into a rat, there were no seizures observed. It was concluded that propylene glycol was the trigger of the seizures to this particular set of rats.

Cardiac puncture was performed to draw blood directly from the heart of a rat that seized (*n* = 1). The animal was anesthetized with isoflurane prior to the cardiac puncture. Sodium levels were 137 mmol/L with potassium at 5.6 mmol/L, due to acidosis from the ion shifts (Table 2). Furthermore, the blood work showed high levels of bilirubin and spherocytes indicating hemolysis (Table 2). The combined findings support a diagnosis of hemolysis and lactic acidosis, which may have led to the seizures, all of which appeared to be a consequence of the propylene glycol administration.

## 3. Discussion

The implication of this report is that propylene glycol, which is a commonly used diluent for water insoluble substances used for parenteral administration, can be acutely toxic to some lines of rats. The objective of this study is not to describe successful treatment of this toxicity, but only to describe that it can occur. The dextrose was initially administered after the onset of seizures because it was thought that the seizures were related to hypoglycemia. Absorption from the oral or rectal mucosa was prompt and seizures ceased within minutes in animals that were going to respond. However, the hypothesis that the seizures were caused by hypoglycemia was later shown to be false, in which case the cause of the seizures was investigated and concluded to be due to the propylene glycol.

**Figure 1:**
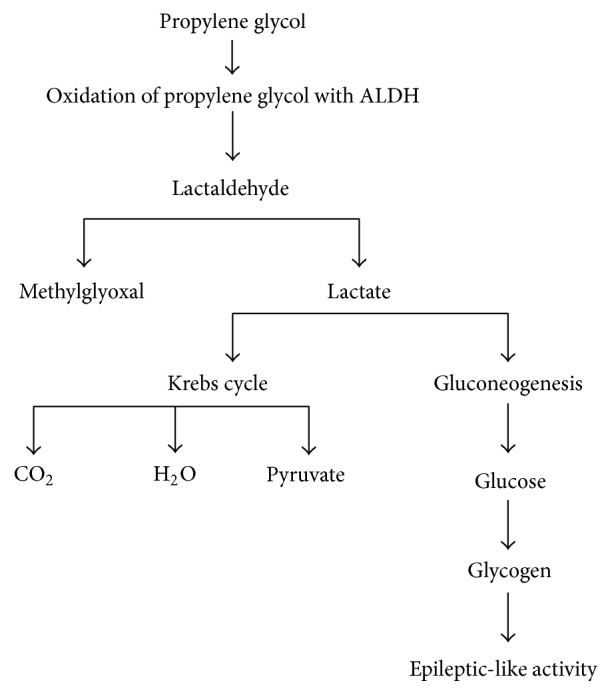
Simplified chart for the metabolism of monopropylene glycol (MPG). Oxidation of propylene glycol by alcohol dehydrogenase (ALDH) leads to primarily lactate production. Epileptic-like activity results if the lactate goes into gluconeogenesis, and astrocytes convert the glucose into glycogen (adapted and summarized from [5, 15]).

The current case of unexpected status epilepticus is thought to be specifically due to an error in the metabolic pathway of propylene glycol. In human adults, propylene glycol is estimated to be eliminated at ~45% by renal route and ~55% through hepatic metabolism through lactate and pyruvate [2, 16]. Similar metabolic pathways are found in rodents such as rats and mice [15, 17]. The mammalian metabolic pathway for propylene glycol (also known as monopropylene glycol or MPG) ultimately results in the formation of pyruvate, carbon dioxide, water, and potentially glucose (Figure 1). Oxidation of propylene glycol by alcohol dehydrogenase (ALDH) leads to lactaldehyde, which gets further broken down into methylglyoxal or lactate [5, 15]. Lactate then enters either the Krebs cycle or gluconeogenesis [5, 15]. The activation of gluconeogenesis results in production of glucose, which astrocytes then convert into polymerized glycogen [5, 15, 18]. The accumulation of gluconeogenesis and glycogen will deprive neurons of energy, which may alter neuronal function and results in epileptic-like activity [18, 19]. We suspect that the gluconeogenesis pathway may have been activated in our rats which resulted in status epilepticus. The suspicion of the gluconeogenesis pathway activation is further supported by the incidents of which administration of 50% dextrose to the seizing rats was able to stop the tremors. Schauwecker found that glucose rescue can diminish the extent of seizure induced cell death [20]. In fact, they believed a deficiency of insulin signaling may have been the factor of seizure induced cell death [20].

Developmental issues and age in both humans and rats may result in complications when using drugs or food containing propylene glycol [1, 3, 14, 21–23]. Administration of pharmaceuticals containing 300 mg–3 g of propylene glycol per day for 14 days to premature newborn babies resulted in rapid progression of hyperbilirubinemia, seizures, and renal failure [22]. Infants who experience seizures due to propylene glycol exposure are believed to likely have undeveloped metabolizing capability and high blood levels of propylene glycol [6, 22]. Thus, if either a human or rodent lacks the capacity to fully break down propylene glycol, negative side effects such as seizures are much more likely.

Toxicity in otherwise healthy humans and rodents of propylene glycol is based on accumulation and concentration. Several adverse effects have occurred following topical, oral, and intravenous administration [1, 21, 24, 25]. Some of these effects include central nervous system toxicity, hemolysis, hyperosmolarity, cardiac arrhythmia, and lactic acidosis [1, 26–29]. In experimental animals, propylene glycol has caused comas, seizures, circulatory collapse, ventricular dysrhythmias, and hypotension [26]. Therefore, it may be detrimental to follow the guidelines of a known lethal dose of 13 g/kg administered intravenously for rats in order to avoid possible adverse side effects [4, 30]. It is important to note that the animals used in our study got a calculated maximum 0.52 g/kg (25 times lower dose). It is also noteworthy to mention that the volume of propylene glycol injected intraperitoneally in the rats described in this case report was no more than 120 *µ*L. Volumes up to 500 *µ*L of propylene glycol have been shown to be safely administered intraperitoneally to rats without any reported seizure like side effects [31, 32].

## 4. Conclusion

In conclusion, this report describes unexpected status epilepticus observed in the latest shipment of Sprague Dawley rats. The seizures were initiated after 300 *µ*L ± 50 *µ*L IP administration of resveratrol in a 40 : 60 propylene glycol/corn oil vehicle solution (equivalent to 0.52 g/kg of propylene glycol). None of the ongoing animals in the study experienced any seizure as side effects to the injections. The concentration and volume of propylene glycol were in accordance with previous toxicology limits. Thus, we conclude this phenomenon is likely due to

1. issues in the metabolic pathway of propylene glycol including (i) missing enzymes for lactate and/or pyruvate breakdown, (ii) activation of the gluconeogenesis pathway, or (iii) toxicity secondary to lactic acidosis,
2. underdeveloped or premature rats who went undiagnosed,
3. predisposition to epilepsy.

The current findings correlate with other rodent and human pathologies to propylene glycol. This report appears to be the first published case of unexpected status epilepticus due to an IP injection containing propylene glycol. Moreover, to our knowledge this is the first reported case in which animals from an ongoing study have spontaneous adverse side effects halfway through a study.

## Figures and Tables

**Table 1:** Summary of animals described within the case report.

Animal number	Evaluation	50% dextrose administration	Outcome
1	Postmortem necropsy	No	Normal, no gross abnormalities
2	Test hypothesis that seizures were due to hypoglycemia	Oral/rectal	Administration of 50% dextrose and diagnosis of hypoglycemia
3	Blood glucose test	Oral/rectal	Animals were not hypoglycemic; seizures were due to compound injected
4	Identifying component of drug, tested diluent only	Oral/rectal	Animals had seizures
5	Identifying component of drug, tested propylene glycol only	Oral/rectal	Animals had seizures
6	Identifying component of drug, tested corn oil only	No	Animals did not have seizures
7	Cardiac puncture for blood test	No	Diagnosis of hemolysis and lactic acidosis which may have led to seizures

**Table 2:** Summary of relevant blood test results from the cardiac puncture from one rat.

Analyte	Results	Units	Reference range	High/low
Sodium	137	mmol/L	146–151	Low
Potassium	5.6	mmol/L	3.8–5.6	High
Magnesium	2.6	mg/dL	3.8–5.5	Low
Total bilirubin	0.3	mg/dL	0.0–0.1	High
Alanine aminotransferase	49	U/L	59–166	Low
Alkaline phosphatase	205	U/L	232–632	Low
Amylase	399	U/L	545–847	Low
Total protein	5.5	g/dL	5.8–7.1	Low
Albumin	3.1	g/dL	3.2–3.7	Low
Globulin	2.4	g/dL	2.6–3.5	Low
Cholesterol	111	mg/dL	50–92	High
Triglycerides	70	mg/dL	101–369	Low
Creatine kinase	797	U/L	113–692	High
Red blood cell count	6.85	×10^6^/*µ*L	7.0–9.0	Low
Hemoglobin	13.6	g/dL	13.7–16.8	Low
Mean corpuscular volume	60.6	fL	49.9–58.3	High
Mean corpuscular hemoglobin conc.	32.8	g/dL	33.2–37.9	Low
Red cell distribution width	18.9	%	10.5–14.9	High
Polychromatophilia	3+	N/A	N/A	Abnormal
